# Characterization of *Helicobacter pylori iceA* and *babA2* virulence genes in dyspeptic patients at a teaching hospital in Ghana

**DOI:** 10.11604/pamj.2024.47.204.39135

**Published:** 2024-04-22

**Authors:** Richard Harry Asmah, Timothy Archampong, Gabriel King, Benjamin Eyison, Andrew Kwablah Teye, Christopher Adjei, Gloria Amegatcher, Ebenezer Krampah Aidoo, Seth Attoh

**Affiliations:** 1 Department of Biomedical Sciences, School of Basic and Biomedical Sciences, University of Health and Allied Sciences, Ho, Ghana,; 2 Department of Medicine and Therapeutics, University of Ghana Medical School, Accra, Ghana,; 3 Department of Medical Laboratory Sciences, School of Biomedical and Allied Health Sciences, University of Ghana, Accra, Ghana,; 4 Department of Medical Laboratory Technology, Accra Technical University, Accra, Ghana,; 5 Division of Pathology, Military Hospital, Accra, Ghana

**Keywords:** *Helicobacter pylori*, endoscopy, *iceA*, *babA2*, Ghana

## Abstract

**Introduction:**

Helicobacter pylori (H. pylori) infection is endemic in Africa. It is a major aetiological factor in the development of peptic ulcer disease and distal gastric cancers. Existing data shows that clinical outcomes are dependent on the virulence of the infecting strain, host´s susceptibility, and environmental factors. In Ghana, a previous study showed that the majority of symptomatic individuals harboured cagA and vacA virulent strains. The main objective of this study was to characterize and assess the significance of other virulence factors, specifically iceA and babA2 in Ghana.

**Methods:**

H. pylori iceA and babA2 genes were investigated in dyspeptic patients at the Korle Bu Teaching Hospital (KBTH), Accra, Ghana. The study employed a cross-sectional design consecutively recruiting patients with upper gastrointestinal symptoms for endoscopy. Nucleic acid was extracted from gastric biopsies using a commercial kit (QIAGEN DNeasy tissue kit). H. pylori babA2 and iceA genes were amplified using extracted deoxyribonucleic acid (DNA) and primers by polymerase chain reaction (PCR).

**Results:**

majority, (71.1%), of the study participants, were H. pylori positive when tested with urease-campylobacter-like organism (CLO). In total, 46 H. pylori urease CLO-positive samples were randomly analyzed by PCR for iceA, of which, 12 (26%) and 7 (15%) were found to have iceA1 and iceA2 respectively. Of the CLO-positive samples, 9 were randomly analysed for babA2 by PCR. Three samples were babA2 positive and 6 were babA2 negative.

**Conclusion:**

in Ghana, although H. pylori is endemic, iceA prevalence is rather low and probably exerts a limited effect on bacterial virulence. Further evaluation would be required, not only to determine association with other virulence factors but more importantly, inter-relationships with wider host and environmental factors that impact on disease pathogenesis.

## Introduction

*Helicobacter pylori* is a microaerophilic, gram-negative bacterium that thrives in the gastric epithelium of a number of vertebrates including humans [1]. Studies have shown that *H. pylori* infection is the most common chronic bacterial infection known to humans [2], with approximately, 50% of the world´s population infected [2]. In Africa, *Helicobacter pylori* infection is endemic with prevalence high across all age groups studied [3]. It is known to be a major cause of peptic ulcer disease and one of the leading independent risk factors for distal gastric cancer. It is the first formally recognized bacterial carcinogen [2]. Additionally, it is also known to be linked to gastric mucosa-associated tissue lymphoma [4,5]. If not treated, the infection or colonization can last a lifetime [2]. However, only a small percentage (10-15%) of the world´s population is affected with *H. pylori* develop disease [6].

Although infection is universally associated with gastritis, the development of clinical and endoscopic disease is dependent on a number of factors, including the virulence of the infecting strain, the susceptibility of the host, and environmental co-factors [7]. A recent study demonstrated the influence of *Helicobacter pylori* virulence factors *cagA* and *vacA* on clinical and endoscopic disease in Accra, Ghana, an endemic sub-Saharan African country [8]. Most biopsies harboured *H. pylori* expressing both *cagA* and *vacA* virulent genes [8], but sparse information is available about the presence of *iceA* and *babA2* genes in patients with dyspepsia in the region.

The objective of this study was to characterize other key virulence factors, *H. pylori iceA* and *babA2* genes to ascertain their significance in dyspeptic Ghanaian patients.

## Methods

**Study design:** this research study received ethical approval from the University of Health and Allied Sciences Research Committee, Ho, Ghana. This study employed a cross-sectional design consecutively recruiting eligible participants. Patient sampling occurred during one endoscopy session per week (Friday mornings), from June - September 2019. Informed consent was obtained from all subjects who met the inclusion criteria by means of either a signature or thumbprint following which the study questionnaire was completed. Recruitment, data collection, and analysis occurred between June 2019 and April 2020.

**Setting:** the study setting was the Korle-Bu Teaching Hospital in Accra which has 2,500 beds and is the main tertiary referral centre in Accra serving the majority of the southern half of Ghana.

**Participants:** patients were eligible if they were medical inpatients or clinic out-patients attending the Endoscopy Unit, Korle Bu Teaching Hospital having been referred with upper gastrointestinal symptoms for endoscopy.

**Variables:** the study questionnaire gathered categorical data on patients´ demographics, bio-data, associated symptoms, lifestyle/dietary habits, signs and endoscopic diagnoses (gastritis, gastric ulcer, duodenal ulcer, gastric cancer). Categorical outcome variables included *H. pylori* status by urease-CLO test and *iceA, babA* positivity by PCR.

**Measurements:** all experiments were performed in accordance with relevant guidelines and regulations. *Helicobacter* status was defined by urease-CLO rapid urease testing on gastric antral biopsies performed at endoscopy. Following upper-gastro-intestinal endoscopy, three systematic gastric antral biopsies per patient were preserved in 0.5 ml DNA-gard solution (Biometrica In, San Deigo, USA).

**Determination of *Helicobacter pylori* status with rapid urease testing:** in the Endoscopy Unit, Korle-Bu Teaching Hospital, a gastric antral biopsy sample following upper gastrointestinal endoscopy was tested by the rapid urease CLO-test (Cambridge Life Sciences Ltd, Cambridge, UK) to determine the presence of *Helicobacter pylori* in samples.

**Genomic DNA extraction:** genomic DNA was extracted from stored tissue samples collected from patients using a QIAGEN DNA mini kit (Qiagen Co Ltd, USA). After extraction, genomic DNA was stored at -20° C until further analysis.

**Molecular analysis of *Helicobacter pylori* virulence genes:** gastric antral biopsies obtained by endoscopy were stored in specimen tubes containing DNA gard solution (Biomatrica, Inc, Oberlin Drive, San Diego, USA) which preserves DNA at room temperature.

**Polymerase Chain Reaction (PCR) analysis of *iceA* gene:** the primers described by Smith *et al*. [9], for *iceA* gene 1 and 2 were used in this work. PCR amplification was performed under the following conditions: 5X PCR buffer (New England, Biolab Inc), 1.0µl of each primer (for *iceA1*; 5' CATTGTATATCCTATCATTAC3' and 5' GTTGGGTAAGCGTTACAGAATTT 3') for *iceA2*, 5'GTTGGGTATATCACAATTTAT3'; 5' TTRCCCTATTTTCTAGTAGGT3'). Five µl of template DNA, and 0.125 µl Taq polymerase, 2 mM MgCl_2_, 0.5 mM dNTPs (dATP, dTTP, dGTP, dCTP) in 25 µl of reaction. The amplification program included an initial denaturation cycle at 95°C for 2 min, 40 cycles at 94°C for 30s, 50°C for 30s, 72°C for 30s, and a final extension cycle at 72°C for 5 min. The product of amplification was 567 and 229/334 bp fragments of *iceA* gene 1 and 2, respectively. After the reaction 10µL of the PCR product was run by electrophoresis at 100 volts using 2% agarose gel (Biopioneer Co, USA) stained with 0-5 ug/mL ethidium bromide (Life Technologies Co, USA) in 1X Tris-acetate EDTA (TAE) running buffer (Biopioneer Co, USA) using 2 µl of blue/orange DNA loading dye (6X) (Promega Co, USA). A hundred nucleotide base pair molecular size marker (Sigma Mo, USA) was run alongside the PCR products on the gel. The gel was photographed using UV illumination (UVIsave gel documentation system, model GAS9200/1/2/3, version 12) and analyzed.

**Polymerase Chain Reaction (PCR) analysis of *babA2* gene:** the primers described by Gerhard *et al*. [10] was used in this work. PCR amplification was performed under the following conditions: 5X PCR buffer (New England, Biolab Inc), 8.5 pmol of each primer (F5'AATCCAAAAAGGAGAAAAAACATGAAA-3' and R5' TGTTAGTGATTTCGGTGTAGGACA-3'), 5 µl of template DNA, and 0.5 U Taq polymerase, 1.5 mM MgCl_2_, 0.2 mM dNTPs (dATP, dTTP, dGTP, dCTP) in 25 µl of reaction. The amplification program included an initial denaturation cycle at 95°C for 3 min, 40 cycles at 95°C for 30s, 57°C for 40s, 72°C for 45s, and a final extension cycle at 72°C for 5 min. The product of amplification was 850 bp fragment of the *babA2* gene. After the reaction, 10 µl of the PCR product was run by electrophoresis at 120 volts using in 2% agarose gel (Biopioneer Co, USA) stained with 0-5 ug/ml ethidium bromide (Life Technologies Co, USA) in 1X Tris acetate EDTA (TAE) running buffer (Biopioneer Co, USA) using 2 µl of blue/orange DNA loading dye (6X) (Promega Co, USA). A hundred nucleotide base pair molecular size marker (Sigma Mo, USA) was run alongside the PCR products on the gel. The gel was photographed using UV illumination (UVIsave gel documentation system, model GAS9200/1/2/3, version 12) and analysed.

**Bias:** in order to address bias, patients with prior *Helicobacter* eradication treatment or proton-pump inhibitor use two weeks preceding endoscopic analysis were excluded as they were potential confounders and likely to reduce or modify *H. pylori* prevalence.

**Study size:** a total of 100 dyspeptic patients were recruited.

**Statistical methods:** data obtained from questionnaires were stored and analysed using Microsoft Excel software. To avoid missing data, data collection was done by trained research staff with follow-up contact details of participants included in the questionnaire. Percentages were used to analyse qualitative variables. Statistical significance was set at p < 0.05.

## Results

**Figure 1:**
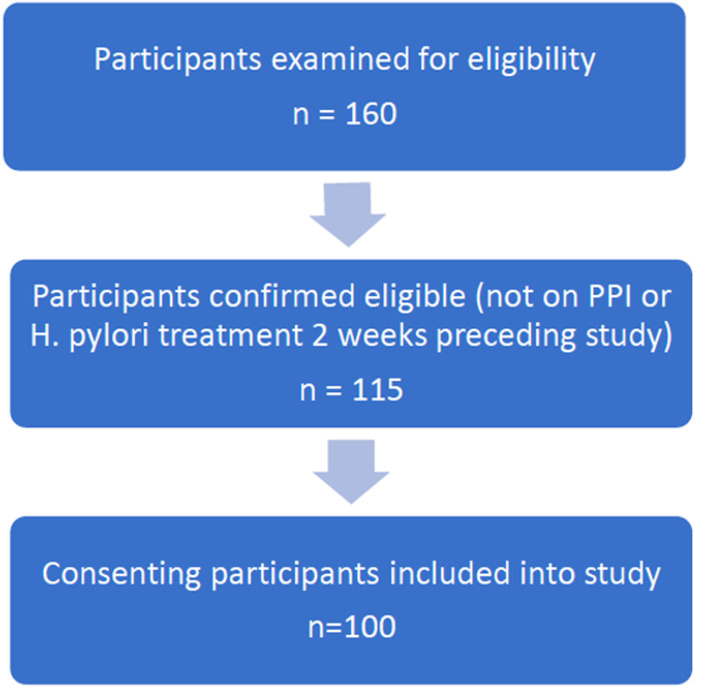
study recruitment flow diagram

**Participants:** approximately eighty patients attended the Endoscopy Unit, KBTH for upper GI endoscopy on a weekly basis during the study period. Ten patients were examined each week for eligibility on Fridays (160 during the study sampling period), of whom 115 patients were confirmed eligible. The main reasons for non-participation in the study included recent antibiotic or proton-pump inhibitor use. Out of the 115 eligible patients, 100 consented and were recruited (Figure 1).

**Descriptive data:** the prevalence of *H. pylori* using the CLO-urease test in the study population was 71% of which 56.3% were male (n=40). The greater majority were in the age range of 31-40 representing 23% followed by the age group 41-50 with 20 participants (20%) (Table 1). Neither age, gender, smoking status, dietary preference, herbal preparation, alcohol intake, nor household composition was associated with *H. pylori* positivity (Table 2). Patients with all the endoscopic diagnoses (gastritis, gastric ulcer, duodenal ulcer, gastric cancer) had an increased prevalence of *H. pylori* (60.5 - 90.1%) in comparison with patients with normal endoscopy (Table 3).

**Table 1:** age distribution of study participants

Age group (yrs)	N	%
Below 20	2	2
21 30	15	15
31-40	23	23
41-50	20	20
51-60	14	14
60-70	13	13
Above 70	13	13

**Table 2:** risk factors for *H. pylori* among the study population

Risk factors	CLO-positive	%	CLO-negative	%	P-value
**Sex**					
Male	40	56.3	13	44.8	0.378
Female	31	43.7	16	55.2	
**Smoking history**					
Smokers	4	5.6	1	3.8	1
Non-smokers	67	94.4	28	96.2	
**Spicy foods**					
Yes	30	42.3	6	20.7	0.065
No	41	57.7	23	79.3	
**Herbal preparation**					
Yes	23	32.4	7	24.1	0.478
No	48	67.6	22	75.9	
**Alcohol**					
Yes	23	32.4	7	24.1	0.478
No	48	67.6	22	75.9	
**Household**					
Compound	44	61.9	13	44.8	0.065
Detached	19	26.8	9	31	
Semi-detached	8	11.3	7	24.2	
**Age**					
<40 yrs	28	39.4	12	41.4	1
≥40 yrs	43	60.6	17	58.6	

CLO: campylobacter-like organism

**Table 3:** endoscopic diagnoses and *H. pylori* status of study participants

Diagnosis	*H. pylori*-positive	%	*H. pylori*-negative	%	Total
Gastritis	26	60.5	17	39.5	43
Gastric ulcer	21	87.5	3	12.5	24
Duodenal ulcer	10	90.1	1	9.9	11
Gastric cancer	4	100	0	0	4
PUD	10	100	0	0	10
Normal	0	0	8	100	8

PUD: peptic ulcer disease

**Figure 2:**
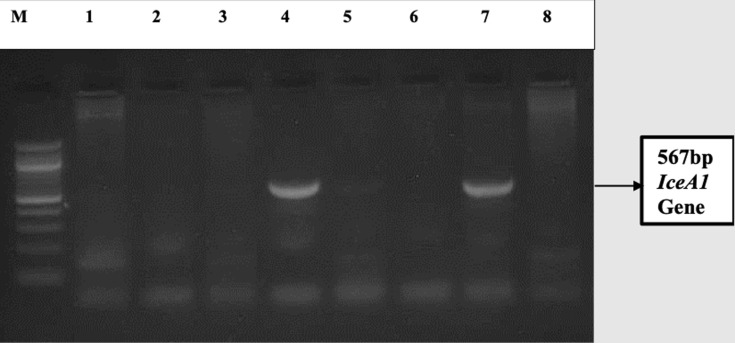
amplicon size of 567 bp obtained from *iceA1* gene PCR analysis (ethidium bromide-stained 2.0% agarose gel electropherogram of amplified *iceA1* DNA fragments (567 bp) with gene primers; lane M is a 100 bp DNA ladder; lane 4 and 7 PCR positives and lane 5 negative control)

**Outcome data:** in total, 46 CLO-positive samples were randomly analysed by PCR to characterize *H. pylori iceA* gene (Figure 2), of which, 12 (26%) and 7 (15%) were found to have *iceA1* gene and *iceA2* genotypes respectively. Figure 2 illustrates the 576 bp PCR amplicon obtained for the *iceA1* gene following 2% gel electrophoresis. Of the CLO-positive samples, 9 were randomly selected and taken through PCR analysis for the *babA2* gene (Table 4). Three samples were *babA2* positive and 6 were *babA2* negative.

**Table 4:** prevalence of *iceA1, iceA2* and *babA2 H. pylori* virulence genes in dyspeptic patients

Genotype	Positive	%	Negative	%	Total
*iceA1*	12	26.1	34	73.9	46
*iceA2*	7	15.2	39	12.5	46
*babA2*	3	33.3	6	66.7	9

## Discussion

In Ghana, there was a high prevalence of infection from *H. pylori*, 71.1%, as previously identified [8]. The pathogenesis of *H. pylori* is orchestrated by a myriad of virulence factors that facilitate colonization, inflammation, and host injury [11]. The *cag* pathogenicity island (*cagPAI*) and vacuolating cytotoxin A (vacA) are undoubtedly some of the most evaluated virulence factors of *H. pylori* [12]. The risk of peptic ulcer disease, pre-malignant gastric pathology (intestinal metaplasia and gastric atrophy) and ultimately distal gastric adenocarcinoma have a higher incidence in patients infected with *cagA*-positive strains when compared with persons infected with *cagA*-negative strains [13,14]. Furthermore, strains containing *vacA* alleles with s1, i1, or m1 sub-type have been demonstrated to have significantly elevated vacuolating activity than those with s2, i2, or m2 and are associated with an increased risk of peptic ulcer disease, pre-malignant lesions as well as gastric cancer [15,16]. A previous study in Ghana, showed that the majority of infected dyspeptic individuals at the tertiary centre harboured *cagA* and *vacA* virulent strains [8].

It is noteworthy that other virulence factors have been implicated in *H. pylori* disease pathogenesis [17]. Specifically, *iceA* has been reported by van Doorn LJ *et al*. [18] as significantly associated with peptic ulcer, with this relationship independent of the *cagA* and *vacA* status. *IceA* has two main allelic forms, *iceA1* and *iceA2* [17]. The expression of *iceA1* has been shown to be upregulated when *H. pylori* adheres to human epithelial cells, with the *iceA1* genotype associated with increased mucosal interleukin (IL)-8 expression and gastric inflammation [19,20]. However, the prevalence and clinical influence of *iceA* varies across populations [17]. The overall prevalence of *iceA1* was significantly higher in Asian countries than in Western countries (64.6% vs. 42.1%), whereas the prevalence of *iceA2* was more prevalent in Western countries than in Asian countries (45.1% vs. 25.8%) [17]. By contrast, *iceA* was clinically significant in Western populations and not Asian countries. Further correlation analysis showed differing relationships with the two isoforms of *iceA, iceA1* being significantly associated with peptic ulcer but *iceA2* rather inversely associated with peptic ulcer [17].

This study was designed to characterize *H. pylori iceA* genotypes in Ghana. Of the 46-CLO positive samples analyzed, 12 (26%) and 7 (15%) were found to have *iceA1* and *iceA2* genotypes respectively (Table 4). *IceA* prevalence in this study in Accra, Ghana was markedly lower than its prevalence in a previous study of 86 dyspeptic South African studies where *iceA1* was detected in 68% and *iceA2* in 80% of all clinical isolates [21]. Genetic analysis of *iceA1* in the South African study demonstrated significant homology (92-95%) with the USA type strain 26695 [21], implying it is likely aligned to Western strains. Another study in Nigeria found *iceA1* prevalence of 94.7% and 86.4% in isolates from duodenal ulcer and non-ulcer dyspepsia respectively [9]. In Ghana, although *vacA* and *cagA* are endemic, *iceA* prevalence is rather low and probably exerts a limited effect on bacterial virulence.

The *H. pylori* genome has several outer membrane proteins (OMP) related genes [13]. Most OMP-encoding genes are preserved in all *H. pylori* strains; however, some may be differentially present across isolates [13]. One of the most widely studied OMPs, *babA* adheres to the fucosylated Lewis b histoblood group antigen on host cells [22]. These adhesion proteins vary in prevalence globally. High prevalence regions include Eastern Asia where all express *babA2* while low prevalence areas were Western and Southern Europe which had rates of 44.0% and 44.6% respectively [23]. Of the CLO-positive samples in this present study, 9 were randomly selected and taken through PCR analysis for the *babA2* gene (Table 4). Three samples were *babA2* positive and 6 were *babA2* negative.

**Limitations:** *babA2* had a low prevalence in this study but given the small number of samples, no further inferences can be made as it is unlikely to be generalizable to the larger population. This study being descriptive, did not provide a correlation between *iceA* and clinical phenotype or *cagA/vacA* status.

## Conclusion

In Ghana, although *H. pylori* is endemic, *iceA* prevalence is rather low and probably exerts a limited effect on bacterial virulence. Further evaluation would be required, not only to determine association with other virulence factors but more importantly, inter-relationships with wider host and environmental factors which can impact on disease pathogenesis.

### What is known about this topic

- *The iceA1 genotype is associated with increased mucosal interleukin (IL)-8 expression and an increase in gastric inflammation;*
- *The prevalence and clinical influence of iceA varies across populations, iceA1 being significantly higher in Asian countries than in Western countries (64.6% vs. 42.1%), whereas iceA2 was more prevalent in Western countries than in Asian countries (45.1% vs. 25.8%);*
- *H. pylori iceA has been shown to be clinically significant in Western populations and not Asian countries*.

### What this study adds

- *Patients with all the endoscopic diagnoses (gastritis, gastric ulcer, duodenal ulcer, gastric cancer) had an increased prevalence of H. pylori (60.5 - 90.1%) in comparison with patients with normal endoscopy;*
- *In Ghana, although vacA and cagA are endemic, iceA prevalence is rather low; of the 46-CLO-positive samples analysed, 12 (26%) and 7 (15%) were found to have iceA1 and iceA2 genotypes respectively*.
